# Endoscopic and clinicopathological patterns of esophageal cancer in Tanzania: experiences from two tertiary health institutions

**DOI:** 10.1186/1477-7819-11-257

**Published:** 2013-10-04

**Authors:** Mabula D Mchembe, Peter F Rambau, Phillipo L Chalya, Hyasinta Jaka, Mheta Koy, William Mahalu

**Affiliations:** 1 Department of Surgery, Muhimbili University of Health and Allied Sciences, Dar Es Salaam, Tanzania; 2 Department of Pathology, Catholic University of Health and Allied Sciences- Bugando, Mwanza, Tanzania; 3 Department of Surgery, Catholic University of Health and Allied Sciences- Bugando, Mwanza, Tanzania; 4 Department of Internal Medicine, Catholic University of Health and Allied Sciences- Bugando, Mwanza, Tanzania; 5 Endoscopic unit, Catholic University of Health and Allied Sciences- Bugando, Mwanza, Tanzania; 6 Cardiothoracic unit, Catholic University of Health and Allied Sciences- Bugando, Mwanza, Tanzania

**Keywords:** Esophageal cancer, Endoscopy, Clinicopathological patterns, Tanzania

## Abstract

**Background:**

Esophageal cancer is one of the most serious gastrointestinal cancer worldwide, owing to its rapid development and fatal prognoses in most cases. There is a paucity of published data regarding esophageal cancer in Tanzania and the study area in particular. This study was conducted to describe the endoscopic and clinicopathological patterns of esophageal cancer in this part of the world. The study provides baseline local data for future comparison.

**Methods:**

This was a retrospective study of histologically confirmed cases of esophageal cancer seen at Bugando Medical Center and Muhimbili National Hospital between March 2008 and February 2013. Data were retrieved from medical record computer database and analyzed using SPSS computer software version 17.0.

**Results:**

A total of 328 esophageal cancer patients were enrolled in the study, representing 25.3% of all malignant gastrointestinal tract tumors. The male to female ratio was 2.2:1. The median age of patients at presentation was 47 years. The majority of patients (86.6%) were peasants coming from the rural areas. Smoking and alcohol consumption were documented in 74.7% and 61.6% of patients respectively. Family history of esophageal cancer was reported in 4.6% of cases. The majority of patients (81.7%) presented late with advanced stage of cancer. Progressive dysphagia and weight loss were the most common presenting symptoms occurring in all patients. The middle third esophagus (58.5%) was the most frequent anatomical site for esophageal cancer followed by lower third (27.4%) and upper third esophagus (10.4%). Squamous cell carcinoma (96.0%) was the most common histopathological type. Adenocarcinoma occurred in 13 (4.0%) patients. TNM staging was documented in only 104 (31.7%) patients. Of these, 102(98.1%) patients were diagnosed with advanced esophageal cancer (Stages III and IV). According to tumor grading, most of tumors were moderately differentiated accounting for 56.1% of cases. Distant metastasis was documented in 43.3% of patients.

**Conclusion:**

Esophageal cancer is not uncommon in this region and shows a trend towards a relative young age at presentation and the majority of patients present late with advanced stage. There is a need for screening of high-risk populations and detecting esophageal cancer at an early stage in order to improve chances for successful treatment and survival.

## Background

Esophageal cancer is one of the most serious tumors worldwide, owing to its rapid development and fatal prognosis in most cases [1]. Worldwide, esophageal cancer ranks eighth in cancer incidence and sixth in cancer mortality [2,3]. The incidence of esophageal cancer varies widely, and certain areas such as northern China [1,3], northeastern Iran [4], and South Africa [5] have very high rates of this disease, with age-standardized incidence rates from 50 to over 100 cases per 100,000 population per year. In contrast, most Western countries have much lower incidence rates of esophageal cancer, from 4 to 10 cases per 100,000 population per year [5].

Epidemiological studies have identified tobacco, consumption of maize contaminated by *Fusarium verticilloides* and nitrosamine as well as human papillomavirus (HPV) infection as risk factors associated with the development of cancer of the esophagus [6-8]. Tobacco and HPV infection have been associated with non-endemic esophageal cancer while the endemic cancer has been associated with maize meal, which is used as staple food, due to contamination by fungal mycotoxins, as well as nutritional deficiencies [9,10]. The risk of esophageal cancer increases with constant exposure to stimulants, hot beverages, alcohol and smoking. It has higher incidence in societies with low economic status, severe malnutrition, low vitamin, fruit and vegetable intake, and also high consumption of alcohol and cigarettes. The unchangeable factors in this cancer include age, sex and hereditary background. In various studies conducted in China, relative risk of patients with a family history of esophageal cancer was around 2.9% of cases [11]. Its prevention is based on early detection and surveillance of precancerous lesions, especially of Barrett’s esophagus, and attention should also focus on modification of changeable risk factors, including tobacco smoking, alcohol abuse, and ingestion of hot and spicy food. The occurrence of esophageal cancer increases with age with the highest incidence in the age group 50 to 70 years. The disease is diagnosed more frequently in male than in female patients with an approximate ratio of 3-to-5:1 [12].

There are two main histological types; squamous cell carcinoma and adenocarcinoma. Worldwide, squamous cell carcinoma is the predominant histological type [13]. The peak age of incidence of squamous cell carcinoma is in the sixth decade in most studies, although adenocarcinoma appears to be commoner in males under the age of 40 years [12]. Worldwide, a higher incidence of esophageal cancer is seen in men with an average 3- to 4-fold increased rate for squamous cell carcinoma and a 7- to 10-fold increased rate for adenocarcinoma compared to women [12,13]. There is racial variation in the histological types with predominance of squamous cell carcinoma in black populations, representing over 90% of all esophageal cancers in Africa [14-16]. Adenocarcinoma is the predominant type in Western countries [13]. In the US, adenocarcinoma of the esophagus is reported to be the most common malignancy with the fastest growing incidence, having increased six times in three decades [13,16].

The middle third of the esophagus is the commonest site for squamous cell carcinoma and the lower third is the commonest site for adenocarcinoma [12,14-16]. Most patients present with progressive dysphagia and weight loss with dysphagia being the most important and the first symptom. As most patients present at advanced stage, mortality is very high and even with operable tumors, postoperative mortality is about 50% [17]. The symptoms of esophageal cancer usually appear 3 to 4 months prior to diagnosis and such symptoms vary depending on the segment of esophagus initially involved. Dysphagia is the most common symptom occurring in more than 90% of cases; weight loss over 5 to 10% of body weight occurs in the major ity of patients and is associated with a worse prognosis [18]. Less common symptoms, such as hoarseness, cough and progressive lesions with invasion to other organs result in symptoms such as hematemesis, hemoptysis, dyspnea and cough secondary to bronchoesophageal and tracheoesophageal fistula [17,18].

The clinical stage of the disease at presentation is important for the outcome of the patient with esophageal cancer. However, the outcome of treatment of esophageal cancer in our environment has been poor because the majority of patients present late to the hospital with an advanced stage and only palliative care is possible. This is partly due to the paucity of published local data regarding this condition and lack of community awareness on the importance of early reporting to hospital for early diagnosis and treatment.

The pattern of esophageal cancer is rapidly changing worldwide. In Western countries, adenocarcinoma of the lower esophagus has overtaken the previously more prevalent squamous cell carcinoma [13]. This observation makes it essential that studies are made periodically in every region to describe the patterns of this disease with the idea to do work on their prevention. It is with this background that the authors were prompted to analyze this problem. This study was conducted to describe the endoscopic and clinicopathological characteristics of esophageal cancer in two tertiary care hospitals in Tanzania and compare our results with what is seen in Western countries. The study provides baseline local data for future comparison.

## Methods

This was a retrospective study of patients with histological diagnosis of esophageal cancer seen at Bugando Medical Center and Muhimbili National Hospital between March 2008 and February 2013. Bugando Medical Centre is situated along the shore of Lake Victoria in northwestern Tanzania. It is a tertiary care and teaching hospital for the Catholic University of Health and allied Sciences-Bugando (CUHAS-Bugando) and has a bed capacity of 1,000. It serves as a referral center for tertiary specialist care for a catchment population of approximately 13 million people in northwestern Tanzania. Muhimbili National Hospital is located in the capital city of Dar es Salaam and is the largest hospital in the country with a bed capacity of 1,500. The hospital serves as a national referral hospital and a teaching hospital for Muhimbili University of Health and allied Sciences and other paramedical staff.

The study population included all patients who presented to Bugando Medical Centre with histologically confirmed esophageal cancer during the period studied. Patients with incomplete data were excluded from the study. The details of patients were retrieved from the computer database of the hospital located in the medical record department and histopathology laboratory. Information was collected using a preformed questionnaire. Included in the questionnaire were questions on sociodemographic data, clinical presentation, anatomical site, gross appearance, tumor, node, metastasis (TNM) staging, histopathological type and grade, and presence of metastasis. The severity of dysphagia was graded as follows: grade 1: normal swallowing; grade 2: difficulty in swallowing solids; grade 3: difficulty in swallowing semisolids; grade 4: difficulty in swallowing liquids; grade 5: difficulty in swallowing own saliva.

The clinical staging was performed using TNM (American Joint Committee on Cancer (AJCC) cancer staging manual); this is a staging system that is an expression of the anatomical extent of the disease based on the extent of the primary tumor (T), absence or presence of and extent of regional lymph node metastasis (N) and absence or presence of distant metastasis (M) [19].

The diagnosis of esophageal cancer was performed by barium swallow and fiberoptic or rigid esophagoscopy and confirmed pathologically by esophagoscopic biopsies. Chest radiographs, abdominal ultrasound, and computed tomography (CT) of the chest and abdomino-pelvic region were performed whenever necessary to exclude distant metastasis. Endoscopic ultrasound (EUS) was not performed due to lack of this facility at our centre. Other routine laboratory investigations included full blood picture, serum creatinine, serum electrolytes and serum liver function tests.

### Statistical data analysis

Statistical data analysis was done using SPSS software version 17.0 (SPSS, Inc, Chicago, IL, USA). Data were summarized in the form of proportions and frequency tables for categorical variables. Continuous variables were summarized using means, median, mode and standard deviation. *P*-values were computed for categorical variables using the Chi-square (*χ*^2^) test and the Fisher exact test, depending on the size of the dataset. The independent Student *t*-test was used for continuous variables. A *P*-value less than 0.05 was considered to constitute a statistically significant difference.

### Ethical consideration

Ethical approval to conduct the study was obtained from the CUHAS/Bugando Medical Centre/MUHAS Joint Institutional Ethic Review Committee before the commencement of the study.

## Results

During the period of study, a total of 1,296 malignant gastrointestinal tract tumors were registered at Bugando Medical Center and Muhimbili National Hospital. Of these, 328 patients, representing 25.3% of all cases, were histopathologically confirmed cases of esophageal cancer and formed the study population. The age of patients at presentation ranged from 24 to 78 years with a median age of 47 years. The modal age group was 41 to 50 years; 158 (48.2%) patients were aged 50 years or below. There were 226 (68.9%) men and 102 (31.1%) women with a male to female ratio of 2.2:1. The difference in mean age between men and women at presentation was not statistically significant (*P* = 0.456). The majority of patients (n = 284, 86.6%) were peasants coming from the rural areas and most of them, (n = 249, 75.9%) were unemployed. The vast majority of patients (n = 292, 89.0%), had either primary education only or no formal education, and more than 80% of patients belonged to low socioeconomic status (Table 1).

**Table 1:** Distribution of patients according to their characteristics

**Patients’ characteristics**	**Number of patients**	**Percentage of patients**
**Age, years**		
≤50	198	60.4
>50	130	39.6
**Sex**		
Male	226	68.9
Female	102	31.1
**Area of residence**		
Urban	44	13.4
Rural	284	86.6
**Employment**		
Employed	79	24.1
Unemployed	249	75.9
**Education**		
Informal/primary	292	89.0
Secondary/tertiary	36	11.0
**Socioeconomic status**		
Low	274	83.5
High	54	16.5
**Smoking**		
Yes	245	74.7
No	83	25.3
**Alcohol abuse**		
Yes	202	61.6
No	126	38.4
**Family history of esophageal cancer**		
Yes	15	4.6
No	313	94.4

The duration of symptoms ranged from 2 to 14 months with a median duration of 4 months. The majority of patients (n = 268, 81.7%) had experienced symptoms for 1 to 4 months by the time of presentation. All the patients presented with progressive dysphagia (graded as shown in Table 2) and weight loss (100%); 249 patients (75.9%) presented with regurgitation, and 102 (31.1%), 45 (13.7%) and 12 (3.7%) patients presented with retrosternal pain, odynophagia and hematemesis, respectively. Symptoms related to distant metastasis were recorded for 135 (41.2%) patients. Smoking and alcohol consumption were reported by 245 (74.7%) and 202 (61.6%) patients, respectively. There was no history of drug addiction. Family history of esophageal cancer was reported by 15 (4.6%) patients.

**Table 2:** Distribution of patients according to the grade of dysphagia

**Dysphagia grade**	**Number of patients**	**Percentage of patients**
1	-	-
2	15	4.6
3	49	14.9
4	141	43.0
5	123	37.5
Total	328	100

The middle third of the esophagus was the most frequent anatomical site for esophageal cancer in 58.5% of patients. This was followed by the lower third of the esophagus and upper third of the esophagus in 27.4% and 10.4% of patients, respectively. The anatomical site was not recorded in 3.7% of patients. On endoscopic examination, most of lesions were ulcerative (40.2%). The median tumor length according to endoscopy and barium swallow was 5 cm (range 4 to 7 cm). The tumor length was ≤5 cm in 40.2% of patients and >5 cm in 185 (52.4%) patients. The tumor length was not documented in 11 (3.4%) patients. Squamous cell carcinoma was the most common histopathological type, occurring in 315 (96.0%) patients. Of these, 34 (10.8%) tumors were in the upper third, 200 (63.5%) in the middle third and 81 (25.7%) in the lower third. Adenocarcinoma occurred in 13 (4.0%) patients, of which 9 (69.2%) were in the lower third and 4 (30.8%) in the middle third. Out of 13 patients with adenocarcinoma, 3 (23.1%) patients had evidence of Barrett’s esophagus. TNM staging was documented in only 104 (31.7%) patients. Of these, 102 (98.1%) patients were diagnosed with advanced esophageal cancer (stages III and IV). According to tumor grading, most of tumors were moderately differentiated, accounting for 56.1% of cases. Distant metastasis was documented in 43.3% of patients (Table 3).

**Table 3:** Anatomical site, macroscopic appearance, histopathological type/grade and metastasis at the time of diagnosis

**Variables**	**Frequency**	**Percentage**
**Anatomical site**		
Upper third esophagus	34	10.4
Middle third esophagus	192	58.5
Lower third esophagus	90	27.4
Not documented	12	3.7
**Tumor length**		
≤ 5 cm	132	40.2
>5 cm	185	52.4
Not documented	11	3.4
**Gross/macroscopic appearance**		
Ulcerative	132	40.2
Infiltrative	106	32.3
Stricture	12	3.7
Polypoid	7	2.1
Fungoid	6	1.8
Mixed	41	12.5
Not documented	24	7.3
**Histopathological type**		
Squamous cell carcinoma	315	96.0
Adenocarcinoma	13	4.0
Tumor grading		
Well-differentiated	104	31.7
Moderately differentiated	184	56.1
Poorly differentiated	40	12.2
**Tumor staging**		
I and II	2	0.6
III and IV	102	31.1
Not documented	224	68.3
**Distant metastasis**		
Yes	142	43.3
No	104	31.7
Not documented	82	25.0

## Discussion

In this review, colorectal cancer accounted for 25.3% of all malignant gastrointestinal tumors seen during the study period. This figure is significantly higher than the 9.5 to 13.3% recorded in previous studies from Nigeria [20,21]. A high figure of 32.5% was recorded in Addis Ababa [16]. Differences in exposure to several risk factors for esophageal cancer may be responsible for this regional variation. Our figure for esophageal cancer in this study may actually be underestimated by the retrospective nature of the study. A better picture of the incidence of esophageal cancer in this region requires prospective comprehensive data collection.

In keeping with other studies [16,20,21], the peak age incidence of esophageal cancer in this study was found to be in the fourth decade of life, which is about a decade or two earlier compared to the findings in developed countries [3,22,23]. The presence of a high number of young patients with esophageal cancer in our setting necessitates family screening since genetic factors may play an important role in the development of this disease. This makes early detection and management an important measure in order to reduce the incidence, and the mortality associated with this disease.

The male predominance demonstrated in this study was in keeping with previous observations reported in studies performed elsewhere [12,13]. However, equal gender distribution was reported in previous studies from Nigeria [14], and Kenya [15]. The male predominance in our study can be explained by the fact that most of the known risk factors for esophageal cancer are related to behavior - smoking and excessive alcohol consumption - of which men are known to be worse consumers than their gender counterparts.

Globally, esophageal cancer has been reported to be more prevalent in people with low socioeconomic status [11]. This observation is reflected in our study where most of patients had either primary education or no formal education and more than 75% of them were unemployed. Socioeconomic class appears to be an independent risk factor in the development of esophageal cancer [11,24]. The vast majority of patients in the present study came from the rural areas located a considerable distance from the study area. A similar observation was reported by others [14,15]. This finding has implications for the accessibility to health care facilities and awareness of the disease.

Several risk factors have been implicated in the geographic variation in the incidence of esophageal cancer [6-8]. Alcohol and tobacco abuse in the etiology of esophageal cancer is well-established [15,25,26]. In our study, history of alcohol consumption and smoking was documented in 61.6% and 74.7% of patients, respectively. These figures are significantly higher than the 19.7% and 1.3% respectively, as reported (in northwestern Iran) by Aledavood *et al.*[26]. It has been estimated that more than 80% of esophageal cancer cases in industrialized countries can be attributed to exposure to these lifestyle choices, either singly or jointly [15,26]. Substantial alcohol intake, especially in combination with smoking, greatly increases the risk of squamous-cell carcinoma but not adenocarcinoma. The joint effect of alcohol and smoking when consumed together are potentiated and the final relative risk is multiplied [11,15,26].

Family history of esophageal cancer has also been shown to be relevant in some high-risk areas. A study in Shanxi Province, China, found that families who have a prior history of esophageal cancer were significantly more likely to have reported a new case during 10 years of follow up [11]. In the present review, family history of esophageal cancer was reported in 4.6% cases, a figure significantly lower than the 43% reported by Dawsey *et al.*[23]. This finding suggests that genetic factors may be playing an important role in the development of this disease in our country. Based on this alarming observation we suggest that screening programs, especially genetic screening programs, should be considered as a main measure for prevention and control of esophageal cancer in this part of the world.

The two most common symptoms present in all our patients were dysphagia and weight loss which is similar to previous studies from within and outside Africa [12,14-16,27]. In this study, all patients presented at an advanced stage with progressive dysphagia and weight loss. Dysphagia and weight loss are so commonly associated that some authors regard them as being pathognomonic [15]. Dysphagia is usually associated with bulky tumors that obstruct the esophageal lumen, impairing its function and causing pain [12,15]. The absence of serosa and the distensibility of the esophagus delay the symptoms of esophageal cancer until the tumor is advanced. This could contribute to the variable duration of symptoms [28]. The pliability of the esophagus is such that dysphagia occurs when the lumen is obstructed by about 75% of the circumference, although a small tumor may cause a tight stenosis through intense fibrosis [15,27,28].

In the present study, the majority of patients presented late with an advanced stage of cancer, which is in keeping with other studies in developing countries [12,14-16]. Most patients in our environment are diagnosed at a late stage because of the asymptomatic development of the disease, with associated poor prognosis. Late presentation in our study may also be attributed to lack of awareness of the disease, low standard of education and lack of accessibility to health care facilities in this region. Late presentation of cases is an area of esophageal cancer care in our centre that requires urgent attention. Detecting esophageal cancer at an early stage contributes to improve chances for successful treatment and thus, for survival.

The location of the tumor within the length of the esophagus varies with the histological type. Squamous cell carcinoma is commonly found in the middle and distal third of the esophagus while adenocarcinoma is more commonly located in the distal third. In this study, the middle third of the esophagus was the most frequent anatomical site for esophageal cancer in over 50% of cases, which is consistent with previous studies [15,16,26]. Our finding is at variant with other studies [29-31], which reported the distal third of the esophagus as the most common site for esophageal cancer. We could not find reasons in the literature for this anatomical distribution pattern.

Squamous cell carcinoma was the predominant histological type of esophageal cancer in this study, accounting for 96% of cases. This is similar to reports from other parts of Africa and India in which over 90% of esophageal cancer are squamous cell carcinomas [14-16,32]. This contrasts with studies from the US in which adenocarcinoma accounted for 81% and squamous cell carcinoma for 17% [12]. In another study among Asian/Pacific Islanders in the US, the rate of esophageal squamous cell carcinoma was 81% higher than in white populations [31]. The predominance of squamous cell carcinoma in this study may be attributed to the high rate of alcohol consumption and smoking in this part of Tanzania. The high prevalence of adenocarcinoma in Western countries reflects the pathophysiology of the tumor, which has been associated with gastro-esophageal reflux disease (GERD). In this study, only three patients showed histological features of Barrett’s esophagus. Although data from Africa is scant, a review of GERD in African literature by several workers have confirmed that overall, there is higher prevalence of GERD and adenocarcinoma in Western countries than in all regions of sub-Saharan Africa and that although urbanization has increased the risk factors associated with GERD, the impact of this is yet to be seen.

In the present study, distant metastasis was documented in 43.3% of cases. A similar distant metastatic pattern was reported in other studies [14-16]. Late presentation in our area in the majority of patients may also be responsible for the high distant metastatic rate. Esophageal cancer in most developing countries, including Tanzania, has a dismal prognosis because of the advanced stage at presentation. The majority of our patients presented with late-stage disease, making them inoperable.

The potential limitation of this study is the fact that information about some patients was incomplete in view of the retrospective nature of the study. This might have introduced some bias in our findings. Also TNM staging was only performed in few patients due to lack of staging facilities such as EUS, which is used for tumor and nodal staging, and the irregular availability of CT.

## Conclusion

Esophageal cancer is not uncommon in this region, shows a trend towards a relatively young age at presentation, and the majority of patients present late at an advanced stage. Lack of awareness of the disease, poor accessibility to healthcare facilities, lack of diagnostic and staging facilities, lack of screening programs, poor accessibility to adjuvant therapy and the high cost of care are among the hallmarks of the disease in this region and pose a great challenge in the management of these patients. Addressing these challenges will help reverse this trend.

## Abbreviations

AJCC: American Joint Committee on Cancer; CT: Computed tomography; CUHAS: Catholic University of Health and allied Sciences; EUS: Endoscopic ultrasound; GERD: Gastro-esophageal reflux disease; HPV: Human papillomavirus; TNM: Tumor node, metastasis.

## Competing interests

The authors declared that they have no competing interests.

## Authors’ contributions

MDM conceived the study, participated in the design and coordination of the study and drafted the manuscript. PLC contributed in the study design, literature search, data analysis, manuscript writing, editing and submission of the manuscript. PFR, HJ, MK and WM participated in study design, data analysis, manuscript writing and editing. In addition, PFR participated in reviewing the histopathological data and MDM, HJ, MK and WM participated in endoscopic examinations. WM supervised the study and contributed in data analysis, manuscript writing and editing. All the authors read and approved the final manuscript.
